# Body mass index and inflammation in depression and treatment-resistant depression: a Mendelian randomisation study

**DOI:** 10.1186/s12916-023-03001-7

**Published:** 2023-09-14

**Authors:** Vasilios Karageorgiou, Francesco Casanova, Jessica O’Loughlin, Harry Green, Trevelyan J. McKinley, Jack Bowden, Jessica Tyrrell

**Affiliations:** 1 College of Medicine & Health, University of Exeter, Exeter, UK; 2 Genetics Department, Novo Nordisk Research Centre Oxford, Oxford, UK

**Keywords:** Mendelian randomisation, Body mass index, Depression

## Abstract

**Background:**

Major depressive disorder (MDD) has a significant impact on global burden of disease. Complications in clinical management can occur when response to pharmacological modalities is considered inadequate and symptoms persist (treatment-resistant depression (TRD)). We aim to investigate inflammation, proxied by C-reactive protein (CRP) levels, and body mass index (BMI) as putative causal risk factors for depression and subsequent treatment resistance, leveraging genetic information to avoid confounding via Mendelian randomisation (MR).

**Methods:**

We used the European UK Biobank subcohort ($n=451,025$), the mental health questionnaire (MHQ) and clinical records. For treatment resistance, a previously curated phenotype based on general practitioner (GP) records and prescription data was employed.

We applied univariable and multivariable MR models to genetically predict the exposures and assess their causal contribution to a range of depression outcomes. We used a range of univariable, multivariable and mediation MR models techniques to address our research question with maximum rigour. In addition, we developed a novel statistical procedure to apply pleiotropy-robust multivariable MR to one sample data and employed a Bayesian bootstrap procedure to accurately quantify estimate uncertainty in mediation analysis which outperforms standard approaches in sparse binary outcomes. Given the flexibility of the one-sample design, we evaluated age and sex as moderators of the effects.

**Results:**

In univariable MR models, genetically predicted BMI was positively associated with depression outcomes, including MDD ($\beta$ ($95\%$ CI): 0.133(0.072, 0.205)) and TRD (0.347(0.002, 0.682)), with a larger magnitude in females and with age acting as a moderator of the effect of BMI on severity of depression (0.22(0.050, 0.389)). Multivariable MR analyses suggested an independent causal effect of BMI on TRD not through CRP (0.395(0.004, 0.732)). Our mediation analyses suggested that the effect of CRP on severity of depression was partly mediated by BMI. Individuals with TRD ($n=2199$) observationally had higher CRP and BMI compared with individuals with MDD alone and healthy controls.

**Discussion:**

Our work supports the assertion that BMI exerts a causal effect on a range of clinical and questionnaire-based depression phenotypes, with the effect being stronger in females and in younger individuals. We show that this effect is independent of inflammation proxied by CRP levels as the effects of CRP do not persist when jointly estimated with BMI. This is consistent with previous evidence suggesting that overweight contributed to depression even in the absence of any metabolic consequences. It appears that BMI exerts an effect on TRD that persists when we account for BMI influencing MDD.

**Supplementary Information:**

The online version contains supplementary material available at 10.1186/s12916-023-03001-7.

## Background

### Effects of BMI and inflammation on depression and treatment resistance

Depression is a highly prevalent mental health disorder, consistently ranked among the top three leading causes of disability worldwide [1]. In people with a more severe presentation, prescription of an anti-depressant medication, coupled with psycho-social interventions, is recommended as first-line management [2]. For research purposes, response can be quantified as a meaningful reduction in a symptom severity scale, such as the Composite International Diagnostic Interview (CIDI) or Patient Health Questionnaire-9 (PHQ9) and may differ among different antidepressant agents [3]. It is recommended that, if a satisfactory symptom reduction cannot be achieved within the first 6 weeks, then treatment can be augmented with other agents, including other antidepressants, lithium and anti-psychotics [4].

An inadequate response to at least two successive antidepressant medications, each administered for at least 6 weeks, is referred to as treatment resistant depression (TRD) and affects at least $7\%$ of those initially diagnosed with depression [5]. Identifying contributors to treatment resistance early could potentially assist prompt management and guide appropriate interventions targeting other pathways that may predispose to treatment resistance. Recent advances in electronic health record analysis have allowed for the definition of a TRD phenotype in very large databases of routinely collected healthcare data, allowing linkage with existing genetic databases [6].

Overweight and obesity have also been shown to predict the development of depression in multiple observational studies. This relationship could be explained by worsening physical health with obesity which may in turn affect mood. A meta-analysis of 15 prospective cohort studies estimated that being overweight was associated with a $27\%$ increase in the odds of subsequently developing depression. There is also evidence for a dose-response relationship, with obese individuals having higher risk for depression [7]. This relationship could be partially explained by social stigma due to the negative perceptions of overweight/obesity in certain cultures [8]. Another dimension of the effect is its potential appearance later in life. A meta-analysis reported a positive association only in adults older than 20 years of age but not in children and adolescents [7]. Recent studies derived a binary classification of overweight (metabolically favourable and unfavourable adiposity) based on metabolic sequelae, namely hyperlipidemia, compromise in liver function, and sex hormone levels [9, 10]. Whilst individuals with favourable adiposity face much less of the commonly described adverse effects of high adiposity, both phenotypes appeared to exert effects of similar magnitude on the risk of multiple depression outcomes [10]. This was interpreted as a predominantly social, rather than biological, effect. Multiple studies have investigated the effects of weight loss on depressive symptoms in people with overweight or obesity. In general, caloric restriction, behavioural training, or supplements were used as interventions to encourage weight loss, and weight loss was found to reduce depressive symptoms in most studies, as collated in a systematic review [11].

One aspect of the downstream metabolic consequences of overweight that is not explicitly captured by the phenotype of unfavourable adiposity is inflammation. Furthermore, evidence from genome wide association studies has suggested genetic variants important for cytokine and immune regulation predict major depressive disorder (MDD) [12]. C-reactive protein (CRP) is a protein synthesised by the liver as part of the inflammatory response. Measurement of CRP in serum is a common part of investigations for inflammatory conditions, e.g. microbial infections and auto-immune conditions. Given a stable general medical status, CRP levels are largely stable and multiple observational studies have investigated its potential utility as a proxy for disease progression in infectious disease [13] and autoimmune conditions. Despite its predictive value, its potentially causative role in driving the pathophysiological course of a disease has been disputed in multiple settings such as coronary heart disease [14, 15]. In depression, recent work has indicated a higher CRP in 102 individuals with TRD compared with treatment-responsive patients and controls [16].

Despite the advantage of large sample sizes and extensive phenotyping that UKB offers, additional care has to be taken to avoid the inherent limitations of observational data. As phenotypes may be correlated due to confounding rather than a true causal relationship, the measurement of observational associations alone may not reflect a causal mechanism [17]. Mendelian randomisation (MR) is an epidemiological approach that employs genetic variants, most commonly single nucleotide polymorphisms (SNPs) as instrumental variables in order to circumvent environmental confounding [18]. By genetically predicting the levels of an exposure such as CRP by a set of relevant SNPs, the proxied levels of CRP reflect a value of CRP that may not be affected by later-life influences that could distort the value (e.g. BMI, smoking, auto-immune conditions). Associations between genetically predicted CRP and an outcome of interest can then much more readily be interpreted as a causal effect [19].

Previous work investigating the causal role of CRP, interleukin-6 (IL-6, major moderator of CRP), BMI and specific symptom dimensions of depression (sleep, appetite, suicidality) used LD score regression and a range of two-sample MR analyses. The results of this study did not find associations of CRP with any of the outcomes but report an association of IL-6 with suicidality [20]. MR has also been used to investigate how BMI and fat mass affects mood outcomes [12, 21–23].

The expansion of genome-wide association studies (GWAS) has lead to the discovery of multiple new significant causal associations. However, it is likely that many are false positives due to pleiotropy, the phenomenon whereby a SNP is an invalid IV due to exerting an effect on the outcome not through the exposure of interest. [24]. Multivariable MR (MVMR) can be used to assess whether an exposure causally influences an outcome *conditional* on a larger set of genetically instrumented exposures [25, 26]. Incorporating additional exposures stops them from acting as pleiotropic pathways and because of this MVMR is seen to be more robust than univariable MR (UVMR). Indeed, a wealth of evidence exists CRP as a downstream consequence of high body mass. For example, a recent GWAS of serum CRP levels on 204,402 individuals found that adjusting for BMI significantly reduced the strength of association between CRP and well known obesity genes (*FTO* [27]*, TMEM18* [28],* ABO*, previously described genes for obesity) [29].

In this paper, we aim to estimate the causal contributions of CRP and BMI on TRD and other depression phenotypes using a combination of UVMR, MVMR and causal mediation analyses. We further investigate whether these highly correlated exposures exert an independent effect on depression phenotypes or if their effect is mediated, and if these relationships are constant across age distributions.

## Methods

### Data sources

We used UK Biobank (UKB) as the primary data source for genetic and phenotypic information. UKB is a prospective cohort study that recruited approximately 500,000 individuals between the ages 37 and 73 from 2006 to 2010 [30]. An extensive, validated questionnaire was completed by all participants gathering information on sociodemographic variables, environmental exposures and behaviours. All individuals were genotyped; specifically, single nucleotide polymorphisms (SNP) genotypes were obtained from the UKB Axiom$^{TM}$ Array (450,000 individuals) and the UKBiLEVE array (50,000 individuals). These data have undergone rigorous quality checks [31]. Despite the public availability of better powered summary statistics, we restricted the analysis to UKB where access to individual-level data allowed for more flexibility in investigating a range of age- and sex-stratified analyses.

### Exposures

We used BMI measurements and serum levels of CRP. Both exposures are associated with depression or TRD in observational epidemiological studies [5, 16, 32, 33]. We hypothesised that low-grade inflammation could be captured by serum levels of CRP [14]. This biomarker is part of the blood biochemistry test performed in UKB and is available in 429,141 European participants. For BMI, we used the baseline measurement taken at study enrolment. Data on 451,052 participants of European ancestry was available. Inverse normalised CRP and BMI were used to provide a more symmetric distribution than their raw values and to simplify the interpretation of resulting causal estimates as the effect of a 1 standard deviation (SD) higher exposure on the outcome risk.

To assess the independent effects of adiposity and inflammation on depression outcomes beyond the traditional BMI measurement, we report a more granular approach where we examined two distinct gentically predicted phenotypes: unfavourable (UFA) and favourable adiposity (FA) [34]. The FA and UFA genetic sub-groupings were defined based on how SNPs that affect body fat percentage are associated with metabolic markers (high-density lipoprotein, sex hormone binding globulin, triglycerides, aspartate transaminase, alanine transaminase) [34]. These two disjoint sets of SNPs (FA = 36 SNPs, UFA = 38 SNPs) were used to separately instrument body fat percentage and to perform the MR analyses described in MR section. A previous study used MR to assess the causal relationship between FA, UFA and depression. It found a differentiation between the two, observing a significant causal effect of FA on depression, and only a modest effect of FA [10].

### Outcomes

Multiple outcomes were derived in UKB participants based on both the mental health questionnaire (MHQ) and electronic health records. Previous works have described in detail the MHQ [35]), where questionnaires covering a range of psychological measurements (depression, anxiety, unusual experience, post-traumatic stress, substance use) were emailed to a subset of UKB participants and were completed by $n=157,366$ individuals. An additional source of information is through linked electronic health records, including general practitioner (GP) visits. Here, we used a subset of $n=230,000$ UKB participants and used codes to classify participants as having been diagnosed with depression. We use five outcomes: (1) GP diagnosis of any of the read codes describing depressive disorders [6]; (2) lifetime MDD defined by the MHQ measurement [35]; (3) PHQ-9 [36] and (4) CIDI [37] depression severity measures (MHQ [35]); and (5) TRD [6]. A previous work in UKB underlined the low accuracy of self-reported depression measures and marked dilution of GWAS signals compared with clinical diagnostic phenotyping [Cai2020]. To guard against such potentially low resolution of the phenotype, we used both clinical diagnoses (GP diagnosis) and questionnaire data that was filled in by the participants, namely CIDI and PHQ9. These two continuous outcomes were filled in by MHQ participants irrespective of diagnosis both measure depression. A notable difference is that PHQ-9 focuses on the current severity of depressive symptoms in the past 2 weeks, whereas the CIDI targets the duration and impact of symptoms.

#### TRD

Linkage of the GP electronic health records and prescription data, enabled coding of TRD with information on antidepressants prescribed and TRD coded when individuals were prescribed at least two different antidepressants for 6 weeks. For the purpose of this study, we define the treatment interval at 6 weeks, which is more conservative than the four-week change encouraged by prescribing guidelines [38]. This conservative threshold helps reduce the likelihood that drug switching was due to side effects, while still allowing for adequate efficacy.

### Statistical analyses

#### Observational associations

As a baseline model, study outcomes were directly regressed on the observed values of the exposures. For continuous outcomes (CIDI, PHQ-9), multivariable linear models were used. For binary outcomes, we used logistic regression. All models were adjusted for age, assessment centre and sex.

#### MR

A series of one-sample MR analyses were conducted within the UKB cohort. *Instrument Selection*: To avoid winner’s curse bias (inflation of effect estimates due to random variation if the same dataset is used for selection and analysis [39]), external GWAS datasets were screened for genome wide significant SNPs (*P*<5×10−8). SNPs were identified that associated with CRP, BMI and MDD in publicly available GWAS studies not overlapping with UKB [12, 40, 41]. For CRP, SNPs reported by the CHARGE study were extracted [29], whilst for BMI, the Locke et al. study was used [41] with a further specification of a European-focused instrument of 73 SNPs as described by Tyrrell and co-authors [22]. Of the 97 reported SNPs in the Locke et al. study [41], we follow Casanova et al. [10] and limit this to European-specific 76 SNPs. Three further SNPs are excluded due to known pleiotropic effects leading to the final 73 SNPs that constitute the instrument. Specifically, the SNPs rs11030104 (BDNF), rs13107325 (SLC39A8), are excluded because of associations with phenotypes that likely influence depression, respectively with regular smoking, with BP and HDL, and with many traits including alcohol, testosterone and cognitive domains. Clumping was performed with a window of 50kb and an $r^2$ of 0.001 was used to exclude all SNPs in pairwise linkage disequilibrium (LD). This ensured our instrument set was comprised of approximately uncorrelated SNPs. For the analysis where all three CRP, BMI and MDD were genetically proxied, a genetic risk score from the 178 Levey et al. variants was used for MDD [42] in order to facilitate the computationally expensive bootstrap procedure with a single instrument that retains as much variance explained as possible.

After extraction of genotype dosages at the individual level, individual LD matrices were constructed. If any non-negligible amount of pairwise LD was observed ($r^2>0.05$) for two SNPs on the same chromosome, the SNP with the largest *p*-value was retained.

### MR designs

**Fig. 1:**
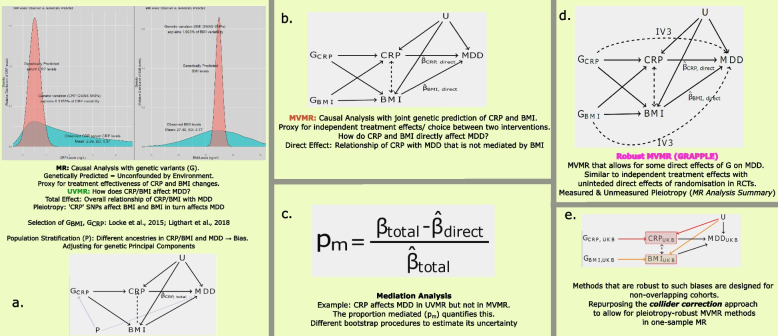
Methods overview. **a** Causal diagram (DAG) representing the assumed relationship between genetic variants for CRP ($G_{CRP}$), measured levels of serum CRP and BMI, and major depressive disorder (MDD). The dashed line between CRP and BMI represents a potential contribution of BMI to CRP levels. **b** DAG for an MVMR analysis that genetically proxies both CRP and BMI ($G_{CRP},G_{BMI}$) enables estimation of the direct causal effect of CRP and BMI on MDD. **c** Estimation of the proportion of the CRP effect mediated by BMI (p$_m$). **d** Robust MVMR to account for unmeasured pleiotropy as well as measured BMI pleiotropy. If some of the genetic variants in $G_{CRP}$ or $G_{BMI}$ affect MDD directly, other than just through changing CRP or BMI levels, the estimated effects will be biased. Robust methods such as MR GRAPPLE protect against this

The different MR analyses reported are visually presented in Fig. 1. All analyses follow the one-sample MR framework, where the exposure (in our case CRP and BMI), genetic variants and the outcomes (depression and TRD) are measured in the same individuals [43]. Within this one-sample framework, we further apply: (a) univariable MR (UMVR) to estimate total causal effects; (b,c) multivariable MR (MVMR) to estimate direct effects and to perform mediation analysis; (d) pleiotropy robust MVMR as a sensitivity analysis.

#### MR analysis summary

The following steps were taken in order to rigorously perform the MR analysis. Firstly, to measure instrument strength, we report the mean *F* statistic for UVMR analyses and the conditional F-statistic [44] for multivariable MVMR analyses. This latter statistic provides a measure of how well a single exposure is instrumented conditionally on the other exposures. Low values indicate the presence of multi-collinearity in the genetically predicted exposures, which leads to weak instrument bias. Secondly, individual-level data UVMR analyses in UKB were carried out using two-stage least squares (TSLS) approach for continous outcomes. For binary outcomes, the second stage linear regression was replaced with logistic regression (otherwise known as two stage predictor substitution). To implement the MVMR analysis, whilst protecting against bias due to weak instruments and pleiotropy, we employed a novel extension of the recently proposed technique of Collider-Correction [45] to the multivariable setting (Fig. 1d, see Appendix for further details). Thirdly, whilst MR methods are generally robust to traditional confounding, they are more susceptible to genetic confounding due to population stratification [46]. To address this, we adjust for the first five genetic principal components in all analyses [47]. This way, we also aim to partly adjust for relatedness; a stricter approach of completely excluding individuals that are related would reduce sample size in an already moderately powered context (loss of $16\%$ of TRD cases). We present the univariable and multivariable analyses in this subset of unrelated individuals. We also use a subset of UKB that includes only individuals of European ancestry ($n=451,025$). Finally, we assessed the extent of heterogeneity amongst causal estimates from different SNPs, a proxy of residual pleiotropy, using the Sargan test [48].

For continuous outcomes, causal estimates reflect the effect of a 1 SD change in the exposure on the outcome. For binary disease outcomes, causal estimates were obtained from a logistic regression and reflect the effect of a 1 SD change on the log-odds of disease.

### Mediation analysis

Recent work has clarified how the results of UMVR and MVMR analyses can be used to assess whether the effect of a single exposure on an outcome is mediated by another exposure [49]. Following Carter et al., we implement our mediation analysis as described below in UKB individual-level data (where *X* and *M* are the exposures of interest (CRP, BMI) and *Y* is the outcome) (Fig. 1c.):

- Estimate the total effect of *X* on *Y* by UMVR, $\hat{\beta }_{total}$.
- Estimate the direct effect of *X* on *Y* $\hat{\beta }_{direct}$ via an MVMR and the indirect effect as $\hat{\beta }_{indirect}=\hat{\beta }_{total}-\hat{\beta }_{direct}$.
- Estimate the quantity $\hat{\pi }_m=\frac{\hat{\beta }_{indirect}}{\hat{\beta }_{total}}$ and its confidence interval via a non-parametric bootstrap of the data in order to test the null hypothesis $H_{0}: \pi _{m}$=0. When $\hat{\beta }_{indirect}$ and $\hat{\beta }_{total}$ have the same sign, $\hat{\pi }_m$ can be interpreted as an estimate for the proportion of the effect of *X* on *Y* mediated via *M*.

#### Bayesian bootstrap for uncertainty quantification and sparse binary outcomes

Carter et al. [49] recommend the use of a standard non-parametric bootstrap in order to provide confidence intervals for $\hat{\pi }_m$. We additionally developed a method to implement Rubin’s Bayesian Bootstrap [50]. Whilst the standard non-parametric bootstrap samples individuals with replacement from the original data, each iteration of the Bayesian bootstrap is always based on the complete data, but the weight each individual receives in the analysis is instead generated from a Dirichlet distribution. Our simulations showed that the Bayesian Bootstrap produces confidence intervals with similar coverage to the non-parametric bootstrap and improved performance for very sparse binary outcomes (Section A.3). For further details, see the Appendix. We used this method to test the hypotheses that the effects of CRP are mediated by BMI and that the effects of CRP and BMI on TRD do not operate solely through MDD, as previously investigated by Maske et al. [51]).

### Sex specific effects and age as a moderator

Our individual level data methods enabled us to perform analyses separately in males and females, formally testing for heterogeneity in causal estimates of BMI and CRP on depression between males and females, using Fisher’s *z*-score. In addition to sex-stratified analyses, we also explored the extent of heterogeneity in causal effects of BMI and CRP across age strata. To achieve this we split the total sample of 451,025 European participants to seven 5-year sub-samples and performed meta-regression to assess whether age was an important predictor of causal effect heterogeneity. (see Appendix A.2 for further information). Results of this analysis are are contained in Table S9.

### Sensitivity analyses

#### Choice of instrument

CRP SNPs have been shown to be highly pleiotropic and affect a range of cardiovascular outcomes and serum lipid traits [40]. In addition to the data-driven pleiotropy-robust methodology described above, we perform a complementary approach of limiting the SNPs used as instruments exclusively to the *CRP* locus. This is based on the hypothesis that SNPs in this specific location represent more biologically relevant CRP variants rather than indirect associations.

We also provide an analysis using the recently proposed cis-MR approach by Patel et al. [52] which uses the complete set of highly correlated SNPs in a single area of biological relevance, instead of limiting the analysis to few independent signals. The argument is that SNPs in close proximity to a gene that codes for a precise molecular target are less likely to affect other phenotypes. We therefore use the CRP genomic region ($1:159712288-4589 \pm 100$kb) and select correlated instruments in the external study [29]. We apply no *p*-value selection threshold. We then use the method of Patel et al. [52] to test the hypothesis that CRP has no effect on the depression outcomes. In this analysis, the exposure data is from the CHARGE study summary statistics [40] and follows the two-sample MR framework.

For BMI, we aimed to locate the Locke et al. instrument to variants that affect BMI through a central mechanism and would hypothetically be more likely to affect depression through other pathways; a tissue enrichment analysis of genes associated with depression showed specific patterns of expression in the brain [42]. We follow a similar approach to Leyden and co-authors [53], employing a dedicated database [54]. The process is presented in detail in Section A.1.

#### Reverse causality

Bias due to reverse causality may emerge when an outcome affects the risk factor, that is a hypothetical causal effect of mood dysregulation on inflammation status and weight. There is abundant clinical literature supporting a longitudinal, potentially bidirectional association of these phenotypes with mood disorders [55, 56] and changes in appetite, eating behaviours and unintended weight gain or weight loss all are included in the diagnostic criteria for MDD [57]. We therefore studied how low mood affects CRP and BMI, using 102 SNPs that associate with MDD [58] to genetically proxy CIDI, one of the MHQ mood questionnaire completed by a subset of UKB participants. Given the previous evidence for depression and BMI sharing a genetic component and as CRP variants also influence BMI, we used Steiger filtering to exclude MDD SNPs that associate more strongly with BMI or CRP rather than MDD, so that the SNP-set $G_{MDD}$ consists of SNPs that predict CIDI more strongly than CRP or BMI [59]. As the instrument strength of $G_{MDD}$ suggested that there may be dilution bias due to weak instruments, a combination of the collider correction approach [45] and the weak-instrument robust MR RAPS approach were also reported [60].

## Results

### Patient characteristics

Table 1 reports the individual characteristics of the UK Biobank participants. The TRD phenotype as previously curated was available for $n=189,917$ controls and $n=2199$ TRD individuals. At the observational level, participants that went on to be diagnosed with TRD had a higher CRP and BMI. The baseline measurements of mood indicated that they scored higher both for CIDI and PHQ9 (Table 1). The proportion of females in the GP depression and TRD groups is higher than in the control group. The majority of individuals have CRP levels that are not consistent with clinically active inflammation, however it seems that there is variability of CRP levels according to depression status, with people with depression and TRD having a 0.55- and 1.1-mg/L higher CRP on average respectively than controls.

Stratifying by this clinical diagnosis status, a statistically significant difference in the PHQ-9 and CIDI scores is observed. Specifically, individuals with an MDD diagnosis and those with TRD exhibited higher scores on both measures compared to those with no diagnosis. This suggests a strong association between clinical diagnosis of MDD/TRD and the severity of depressive symptoms as measured by the PHQ-9 and the presence of depression based on the CIDI. Using a single diagnostic cutoff for a CIDI value at 8 [61], we observed that, among individuals with no diagnosis, a total proportion of 2.08$\%$ met the criterion for MDD, whereas for those with a GP diagnosis of MDD, this proportion was 24.71$\%$, and among individuals diagnosed with TRD), the proportion was 56.17$\%$. These findings suggest that there is a strong correlation of clinical diagnosis status and questionnaire responses.

**Table 1:** Individual characteristics

	Controls	GP-based depression cases	TRD cases	Group comparison
*N*	173,786	18,330	2199	
Age	57.42 (±8.11)	56.11 (±7.95)	56.43 (±7.82)	224.6 ($<\!\!10^{-10}$)
$\%$ female	0.485	0.636	0.724	1804 ($<\!\!10^{-10}$)
BMI	27.38 (±4.67)	28.26 (±5.37)	29.41 (±5.96)	359.1 ($<\!\!10^{-10}$)
CRP (mg/L)	2.54 (±4.35)	2.99 (±4.57)	3.64 (±5.42)	109.7 ($<\!\!10^{-10}$)
CIDI_MDD^a^	2 (±2.84)	5.45 (±2.19)	6.63 (±1.68)	3290 ($<\!\!10^{-10}$)
PHQ9^a^	2.11 (±2.89)	4.52 (±5.09)	9.06 (±6.83)	2240 ($<\!\!10^{-10}$)

Mean (±SD)

^a^ CIDI and PHQ9 were measured in a different, partly overlapping subset of UKB participants ($n=146,067$)

Comparisons across groups are performed with analysis of variance (ANOVA) tests, and *F*-values and *p*-values are reported. A $\chi ^2$ test was used to compare the proportion of females across the three groups

### Univariable and multivariable MR

#### BMI

**Fig. 2:**
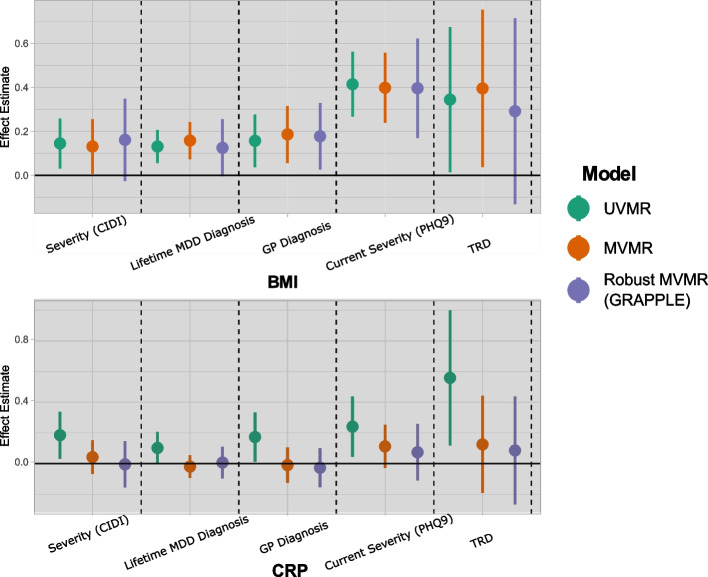
Effects of BMI and CRP on various depression-related outcomes as measured by univariable and multivariable MR models. The CRP effect is measured by using 45 CRP SNPs as instruments. UV, univariable MR; MV, multivariable MR; GRAPPLE, robust multivariable MR with MR GRAPPLE; CIDI, Composite International Diagnostic Interview; PHQ9, Patient Health Questionnaire-9; TRD, treatment-resistant depression

Figure 2 presents the causal estimates for BMI on a range of depression outcomes. In the UVMR analyses of BMI in green, all estimates suggest a robust causal effect, with $95\%$ confidence intervals excluding the null. A 1 SD increase in genetically proxied BMI was associated with 13.9$\%$ (95$\%$ CI$_{OR}$: 8.3$\%$, 22.1$\%$) higher odds of a lifetime diagnosis of depression, 19.7$\%$ (5.1$\%$, 32.3$\%$) higher odds of a GP diagnosis of depression, and 41.9$\%$ (2.0$\%$, 95.4$\%$) higher odds of TRD (Fig. 2). In the MVMR models (in red), this pattern persists, with the analyses with external weights [40, 41] agreeing. The repeat analysis under a stricter approach of completely excluding related individuals similarly supports that BMI has a positive effect on all outcomes (Fig. S1). In the sex-stratified analysis in females (Fig. S2), there is evidence for a causal effect of BMI on all outcomes except for TRD whereas in males, a robust effect is observed only on PHQ9 (severity). Although the BMI point estimates appear larger in magnitude for females, the *z*-test does not suggest statistical significance at the $95\%$ level (Table S1).

Repeating the analyses in each 5-year age stratum, there was an attenuation of the causal effect of BMI on PHQ9 with age ($\beta _{age}=-0.025$ PHQ9 total units per, $p=0.011$) (Fig. S3). This effect was also nominally significant for a non-linear trend ($\beta _{age^2}=-0.22,$ $p=0.0397$, Table S2). The heterogeneity statistics indicate a better fit for the model that includes age for this comparison ($Q_{diff}=6.494,p_{Q_{Diff}}=0.011$). The evaluation of instrument strength in specific groups suggested a lower strength of association in the 69–74 age group (Table S3).

#### CRP

In UVMR, CRP displays an effect on all outcomes (Fig. 2). A 1 SD increase in genetically proxied CRP was associated with 12.7$\%$ (1.0$\%$, 22.3$\%$) higher odds of a lifetime diagnosis of depression, 20.9$\%$ (95$\%$ CI: 2.0$\%$, 40.5$\%$) higher odds of a GP diagnosis of depression, and 63.2$\%$ (13.9$\%$, 146.0$\%$) higher odds of TRD (Fig. 2). In the sex-stratified analyses, strong causal estimates are present for GP diagnosed depression and TRD in females only. All these associations do not persist in the MVMR analyses, where upon jointly predicting BMI and CRP in MVMR models, the estimated effect moves close to the null and significance is lost (Fig. 2). Of note, the consistency of the results remains unchanged even when only unrelated individuals are considered, as illustrated in Fig. S1. The analysis with external weights suggests more modest CRP effects (Fig. S4). There does not seem to by a clear modification of the estimated effects by age but there seems to be heterogeneity on the age-specific effects on GP diagnosis (Fig. S3).

**Fig. 3:**
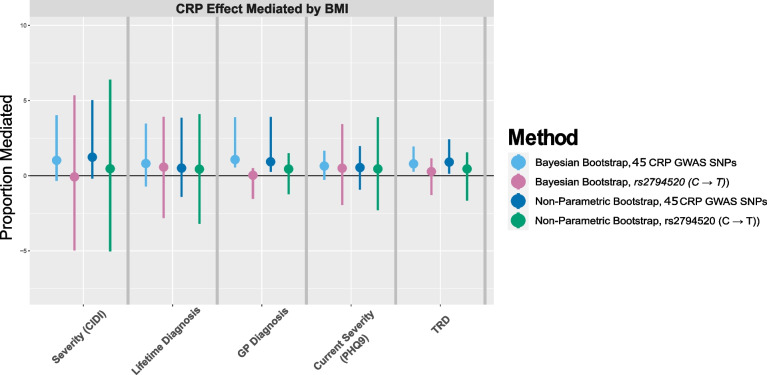
Proportion of CRP effect mediated by BMI, defining the CRP effect with two different instruments (45 CRP SNPs, rs2794520 (C $\rightarrow$ T)) in the *CRP* region). Two methods of bootstrapping are used to estimate the uncertainty (Bayesian bootstrap, non-parametric bootstrap). CIDI, Composite International Diagnostic Interview; MDD, major depressive disorder; PHQ9, Patient Health Questionnaire-9; TRD, treatment-resistant depression

In Table S7, applying the Sargan test revealed significant heterogeneity for all analyses. Therefore, these analyses could be potentially affected by at least one SNP exerting a pleiotropic effect. The reported estimate standard errors account for this heterogeneity, and the estimates themselves are valid under the assumption that the pleiotropy is balanced. A repeat test with the CRP-BMI pair of exposures in an MVMR model indicates that heterogeneity is likely even in the joint model, motivating a pleiotropy-robust method as a more appropriate modelling choice (Fig. 3).

### Sensitivity analyses

#### Favourable and unfavourable adiposity

**Fig. 4:**
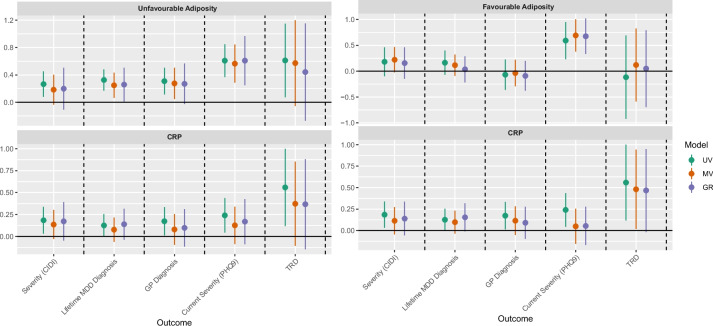
Forest plot for the effect estimates of favourable and unfavourable adiposity and CRP on depression outcomes. CIDI, Composite International Diagnostic Interview; MDD, major depressive disorder; PHQ9, Patient Health Questionnaire-9; TRD, treatment-resistant depression

In Fig. 4, we substituted genetically predicted BMI with genetically predicted UFA and FA, and repeated the assessment of how these affect the outcomes and how the effect of CRP changes. Unfavourable adiposity appears to influence all outcomes, while CRP showed an effect on TRD, PHQ9, and CIDI. FA appears to affect only severity of depression (PHQ9). In the multivariable models, we found that the point estimate of CRP remained relatively stable, indicating a consistent association with the outcomes. In summary, we see a retained effect of adiposity on depression with these more granular phenotypes and an attenuating effect of CRP, as with BMI but more subtly.

#### Robust MVMR

Results for the pleiotropy-robust GRAPPLE implementation of MVMR are presented in Fig. 2 (purple). This method simultaneously accounts for weak instrument bias, imbalanced pleiotropy (via penalisation of outliers) and sample overlap [62]. The results appear to be largely concordant with those of the MVMR, with slightly lower precision and lower magnitude of effects. In this analysis, BMI is associated only with a GP diagnosis of depression and PHQ9. There was insufficient evidence to confirm an effect of CRP with any of the outcomes in the multivariable models.

#### *CRP* gene-specific instrument and tissue-specific BMI SNPs

Specifying the search for valid CRP instruments in the area around the *CRP* gene, 194 variants were identified in the selection sample [29]. After clumping, four were retained as independent (*rs11585798*,* rs2794520*,* rs3934775*,* rs12727193*) and one (*rs2794520*) was strongly associated with CRP ($\beta$ (SE): − 0.182 (0.004) with *T* and *C* as the effect and non-effect alleles, $p=1.2 \times 10^{-305}$). This SNP was carried forward for the analyses in UKB. As in the selection study, *C* carrier status was associated with lower CRP serum levels, more strongly in females ($\beta$ (SE) − 0.172 (0.002) in all, -0.185 (0.003) in women, − 0.158 (0.003) in men); Fisher’s $z=6.01$, $p_{diff}<1.9 \times 10^{-9}$). Using only this SNP as an instrument, UVMR indicates a negative association of CRP with a GP diagnosis of depression ($-0.155 \;(0.07)$). The sex-specific analysis implied a stronger effect in males ($-0.308 \;(0.133)$) and an effect in males only for PHQ9 reaching statistical significance at the 5% level. ($-0.31 \;(0.136)$).

In the MVMR analysis where we also proxy BMI with 73 SNPs, CRP is judged to negatively influence GP diagnosis of depression ($-0.151 \; (0.051)$); this is independent of BMI. This effect is also observed in males ($-0.211 \; (0.089)$), whereas in females it does not reach statistical significance at the 5% level ($-0.113 \; (0.062)$). In the robust MVMR analysis, a statistically significant negative effect was estimated ($-0.144$ (0.055)).

#### Alternative instruments

Regarding CRP, the focused search in the *CRP* genetic region yielded 194 SNPs. Clumping significantly restricted the available variants and only one variant was retained (*rs2794520*). Using the recently proposed approach of Patel et al., it was possible to retain all 194 variants, extract them at the individual allele dosage level and decompose them in independent genetic signals [52]; namely, the variants presented in Fig. S5 were projected in 10 principal components which were then used for MR inference. The results are shown in Table S5. Although the estimates were more precise than those reported in “*CRP* gene-specific instrument and tissue-specific BMI SNPs” section, their magnitude was lower and consequently none surpass the conventional significance threshold.

The tissue-specific MR analyses are presented in Section A.1. Of the 73 BMI SNPs, 23 were retained as being preferentially expressed in brain-related tissues and 31 others that were mapped to coding regions were expressed in the periphery. Similar estimates were obtained for all outcomes. For TRD, UVMR and MVMR suggested a positive effect of BMI on depression only when the peripherally focused instrument was used ($\beta _{UVMR}(CI):0.722(0.246,1.123)$, $\beta _{MVMR}(CI):0.745(0.251,1.229)$). In contrast, the UVMR and MVMR estimates from the instrument that included brain-expressed genes failed to reject the null ($\beta _{UVMR}(CI):0.139(-0.472,0.750)$, $\beta _{MVMR}(CI):0.136(-0.481,0.752)$). Both instruments provided similar results for all outcomes including TRD in the pleiotropy-robust MR GRAPPLE method.

#### Reverse causality assessment using UVMR

Applying the Steiger filtering routine for BMI, 16 of the 102 SNPs reported by Howard et al. [58] were excluded. For the remaining 86 SNPs, instrument strength for the genetic prediction of CIDI was estimated at $F_{stat}=5.337$. In 2SLS, the causal effect of genetically predicted CIDI on BMI was estimated as $\beta$ (SE): 0.077(0.016), but was likely to be affected by weak instrument bias. Using MR-RAPS, the uncertainty in this result increased substantially ($\beta$ (SE):0.074(0.045). For CRP, Steiger filtering indicated that there were 27 SNPs that predict a larger proportion of the CRP variance compared with the CIDI variance. In the remaining 75 SNPs ($F_{stat}=4.132$), an association of genetically proxied CIDI with CRP was observed ($\beta$ (SE): 0.080 (0.020)). Applying MR-RAPS as above, the resulting estimate did not maintain significance at the $95\%$ level ($\beta$ (SE): 0.101 (0.053)).

#### Mediation analysis

The results for the mediation analysis are presented in Fig. 3. When using the discovery set of CRP SNPs ($n_{CRP}=45$), the effect of CRP on GP diagnosis and TRD appears to be mediated by BMI ($107.82\% (56.1\%, 357.02\%)$ and $78.87\% (33.35\%, 197.23\%)$, respectively). For the ever depressed outcome, a similar magnitude was observed but the CIs do not indicate significance at the $95\%$ level ($82.87\% (-208.39\%,250.43\%)$) (Fig. 3). In the sex-specific mediation analysis, BMI appears to mediate the effect of CRP on TRD and GPD in females and on the ever depressed in males (Fig. S6). With the BB method, the CIs for $\pi _m$ are somewhat narrower for TRD. In all methods, the median and 2.5th and 97.5th quantiles of the distribution of the bootstrapped estimates were used to estimate $\pi _m$ and CIs, therefore some asymmetry around $\pi _m$ is observed.

As discussed above, there is a concern for residual pleiotropy in this particular set of SNPs. Since 2SLS models are used throughout and CRP SNPs are known to be highly pleiotropic (“Robust MVMR” section), the estimates with these 45 SNPs may be biased. In the multivariable MR, this is partly alleviated, however there is a possibility of other pathways not related to BMI affecting the outcome. As a result, the estimates of the proportion mediated may be distorted with the full set of 45 SNPs. In a repeat mediation analysis with only one SNP in the CRP locus used to genetically proxy serum CRP levels (*rs2794520*, “*CRP* gene-specific instrument and tissue-specific BMI SNPs” section), the results are not significant at the $95\%$ level (green lines, right panel, Fig. 3).

In the mediation analysis of genetically proxying CIDI and assessing how the CRP and BMI estimates change in a MVMR model with all three as exposures, relatively imprecise results were obtained. This is possibly due to a lack of power ($n=58,586$ individuals with a GP record, follow-up MHQ measurements and baseline CRP and BMI, of whom 514 TRD cases). As expected, CIDI appeared to contribute to TRD in the full model (0.757 (0.382, 1.132)), suggesting that a unit increase in CIDI doubles the odds of TRD independently of CRP or BMI status. In this model, BMI and CRP were positively and negatively associated with TRD ($+0.533 (-0.203, 1.268)$ and ($-0.093 (-0.434, 0.247)$, respectively). In comparison, the corresponding estimates in the univariable model were slightly larger in magnitude ($+0.567 (-0.121,1.255)$ and $-0.127 (-0.459,0.206)$). The mediation analysis results suggest that the independent BMI and CRP effects (Fig. 2) are not mediated by CIDI (p$_{m, CRP}$=$0.231 (-3.372, 4.041)$, p$_{m, BMI}$=$0.288 (-2.375, 3.221)$).

## Discussion

In this study, we aimed to investigate the causal effects of CRP and BMI on depression-related phenotypes, including TRD, using various MR methods to overcome a series of methodological issues [45, 52, 62]. We show that apparent significant findings from univariable MR analyses of CRP do not persist in MVMR analyses adjusting for BMI, indicating that BMI may be the primary driver of the observed association between CRP and depression outcomes. We also found evidence for BMI exerting a positive causal effect on investigated outcomes, particularly in women, and that age may attenuate the effect on severity of depression. We also describe two methodological improvements, an application of multivariable pleiotropy-robust methods in one-sample data and a more precise measurement of mediated effects in sparse binary outcomes.

We find evidence further supporting the influence of BMI on depression, with larger effect sizes observed in women. An attenuation of this effect on severity of depression was also found. Although the sex difference was not statistically significant, it aligns with previous research by Tyrrell et al. [22] on the role of BMI in depression. Our findings suggest that BMI directly influences depression beyond the inflammatory consequences of overweight, addressing a limitation in prior studies. The influence of social processes, including social stigma, may play a crucial role in the relationship between BMI and depression. Our results are in line with a recent work that suggested a causal relationship between trauma and MDD, that is independent of BMI [63].

Sensitivity analyses using brain-specific and periphery-specific instruments yielded similar effects of BMI on depression measures, indirectly highlighting a social aetiology that is independent of metabolic or inflammatory status. For resistance to antidepressant treatment, peripherally expressed BMI SNPs indicated a positive effect on TRD, although uncertainty in the estimates limits strong conclusions. This could be related to a differential metabolic breakdown of antidepressants in those with a periphery-driven difference in adiposity. Future works could also look into more detailed liver and kidney markers, considering their influence on antidepressant metabolism and effectiveness.

Discrepancies across different models were seen for CRP. While a positive effect on depression outcomes was found in the main analyses, this did not persist when jointly estimating BMI and CRP effects for all depression outcomes, and additionally when using cis-MR. Although conditional instrument strength is adequate in these models [26], residual pleiotropy (i.e. other than through CRP or BMI) was still a concern. Indeed, using pleiotropy robust MVMR provided results where the null could not be rejected. Another way to address this issue is to use a stricter selection of variants based on their biologically plausibility as instruments. For example, Kappelmann et al. [20] assessed the role of CRP and IL-6 in individual symptom domains of depression, using a range of instrument selections rules. Another dimension that could explain the indirect effects of inflammation is early or later-life traumatic events [63]. In an MR study, an inflammatory marker that is presumably more sensitive to chronic upregulation, glycoprotein acetyls, also exhibited no evidence for an effect on depression [64].

In the causal analyses for the reverse pathway (from depression outcomes to BMI and CRP), a significant proportion of SNPs were excluded in Steiger filtering as they appeared to be more strongly associated with CRP or BMI. Low predictive capacity of the 102 SNPs was observed for the mood questionnaire that quantified depressive symptomatology. We bypassed this issue by implementing a weak-instrument and pleiotropy-robust method [60], which suggested that there is no effect of depressive symptomatology to BMI or CRP. The estimates were in line with those in 2SLS but uncertainty was larger.

Contextualising our results clinically, BMI reduction appears to have a positive impact on mood, particularly in females and younger individuals. It appears that potential harmful effects of inflammation on mood do not persist when we account for BMI, suggesting that clinical studies of anti-inflammatory medications as adjuncts for MDD should consider the role of body weight. Similar findings emerged in the context of the more severe experience of TRD, indicating the need to consider eating patterns and how these relate to mood. Overall, we recognise that in the care for MDD, other factors and psychological suffering take precedence and it is important to prioritise interventions.

Strengths of our study include the adjustment of novel MR methods to address the issues of mediation and pleiotropy. We employed a range of cutting-edge MR methods including GRAPPLE [62] and cis-MR [52], and overcome a series of methodological issues, including the application of pleiotropy-robust methods in MVMR through an extention of the Collider-Correction algorithm [45]. We also assess different methods of precise mediation analyses and provide a new method that performs better in cases of sparse binary outcomes (the Bayesian bootstrap).

Our study has limitations to be considered. A limitation of the BMI and CRP phenotypes is that they do not fully capture the metabolically harmful aspects of adiposity and the concept of inflammation respectively. Although we observe similar effects when we proxy favourable and unfavourable adiposity, there still is room for improvement for the inflammatory aspect. As data on proteomics is becoming available at a large scale, more refined analyses will be feasible. Assortative mating plays a significant role in the genetic correlation of depression and various psychiatric disorders [65] and it could ostensibly also account for some of the observed effect of body mass and depression outcomes as the BMIs of partners also tends to be phenotypically correlated. Both adiposity and depression are moderately heritable [66, 67] and it has been shown that bias due to assortative mating increases for more heritable phenotypes [68]. This distorts heritability estimates [69] and, to a degree, the magnitude of the effects of the SNPs used as instruments. Future studies of BMI and depression could control for assortative mating by dedicated techniques, such as analyses with parent-offspring trios [68].

## Conclusion

We have applied a range of methods that provide causal estimates in a methodologically robust fashion, that suggest that the effect of CRP on depression outcomes is likely attributable to the influence of BMI. It is not feasible however to definitively exclude the influence of inflammatory status on mood. Immune dysregulation is a wider, more complex phenomenon and we have limited our investigations to serum CRP results and related genetic variants. Further research is needed to understand if these generalise more broadly to other inflammatory mediators.

### Supplementary information

**Additional file 1:** **Appendix** [70–74]. **Figure S1.** Estimated effects of CRP and BMI on a range of depression-related outcomes in a subset of unrelated individuals (n = 52, 510 completed MHQ and contributed to PHQ9 and CIDI, n = 165, 378 with CPRD data linkage that contributed to GP Diagnosis of MDD and TRD. **Figure S2.** Sex-Stratified Analysis for all outcomes reported in Fig. 2. Estimates from univariable MR (UV), multivariable (MV), and pleiotropy-robust multivariable MR (GRAPPLE) are reported for females and males separately. **Figure S3.** MR Analysis with external weights for BMI [Locke2015] and CRP [40]. **Figure S4.** Age as a Moderator of the causal associations of CRP and BMI with mood outcomes. In the visualisation of the meta-regression slope, if the intercept falls within the confidence region of the age slope, then the result is not statistically significant. **Figure S5.** SNP-CRP associations and SNP-depression associations for n = 194 SNPs in LD. These genetic associations are then projected to independent genetic components (cis-MR, [51]). **Figure S6.** Proportion of CRP effect mediated by BMI in males and females. The CRP effect is defined by two different instruments. cisMR: CRP effect is estimated with one SNP as instrument (rs2794520). **Figure S7.** Tissue Expression for SNPs in Locke et al. Transcript per million (TPM) data, scaled per gene, are presented. Ordering follows the sum of scaled TPM across brain regions. **Figure S8.** Estimates of the Effect of BMI on the depression outcomes when two different tissue expression-informed instruments are used. In the top panel, the top 20 SNPs of genes that are predominantly expressed in the brain are shown (Brain); in the bottom panel, genes that are expressed in the periphery constitute the instrument. **Figure S9.** Assumed Data Generated Mechanism for two exposures (X1, X2). Some of the variants that associate with X1 also affect Y directly, thereby violating the third IV assumption. **Figure S10.** Estimation of the causal effect of two exposures X1 and X2 in the presence of weak instrument bias and pleiotropy. The targets of the estimation of $\beta _{X1} = 1$ and $\beta _{X2} = 0.5$ are visualised with grey dashed lines. CFS: Conditional F statistic; CC: Collider Correction; 2SLS: Two-Stage Least Squares; X1, X2: Exposures 1 and 2 in Figure S9. **Figure S11.** Directed Acyclic Graph. The exposure X and the mediator M exert two independent effects on Y. Genetically proxying only X can result in an inaccuracy in the estimation as $\beta _{XY, Univariable}$ will be capturing the total effect ($\beta _{X2} + \beta _{XM} * \beta _{MY}$). **Figure S12.** Uncertainty in estimating the proportion of mediated effects, simulation Results. CFS: Conditional F statistic for the exposure X in Figure S11; CFS$_M$: Conditional F statistic for the mediator M. Error bars in the coverage and power plots represent the Monte Carlo error for $s = 6000$ simulations. BB : Bayesian bootstrap; $mediat\_package$: implementation with the R mediation package; norm: non-parametric bootstrap. **Figure S13.** Simulation Results ($\pi _m$ and 95% Confidence Intervals) for Increasing Prevalence of Binary Outcome.

## Abbreviations

BMI: Body mass index
CRP: C-reactive protein
CIDI: Composite International Diagnostic Interview
FA, UFA: Favourable, unfavourable adiposity
GP: General practitioner
GWAS: Genome-wide association study
MDD: Major depressive disorder
MR: Mendelian randomisation
MVMR: Multivariable Mendelian randomisation
PHQ9: Patient Health Questionnaire-9
SNP: Single nucleotide polymorphism
TRD: Treatment-resistant depression
UKB: UK Biobank
UVMR: Univariable Mendelian randomisation
